# Comparability of a provisioned device versus bring your own device for completion of patient-reported outcome measures by participants with chronic obstructive pulmonary disease: quantitative study findings

**DOI:** 10.1186/s41687-022-00521-3

**Published:** 2022-11-26

**Authors:** Stacie Hudgens, Louise Newton, Sonya Eremenco, Mabel Crescioni, Tara Symonds, Philip C. G. Griffiths, David S. Reasner, Bill Byrom, Paul O’Donohoe, Susan Vallow, the Patient-Reported Outcome (PRO) Consortium and Electronic Clinical Outcome Assessment (eCOA) Consortium

**Affiliations:** 1 Clinical Outcomes Solutions, 1820 E River Rd, Suite 220, Tucson, AZ 85718 USA; 2 Clinical Outcomes Solutions, Folkestone, UK; 3 Critical Path Institute, Tucson, USA; 4 College of Public Health, University of Arizona, Tucson, USA; 5 Imbria Pharmaceuticals, Boston, MA USA; 6 Signant Health, London, UK; 7 Medidata Solutions, London, UK; 8 Novartis Services Inc, East Hanover, NJ USA

**Keywords:** Bring your own device, BYOD, Patient-reported outcome, PRO, COPD

## Abstract

**Objective:**

To quantitatively compare equivalence and compliance of patient-reported outcome (PRO) data collected via provisioned device (PD) versus bring your own device (BYOD).

**Methods:**

Participants with stable chronic obstructive pulmonary disease (COPD) completed the EXAcerbations of Chronic Pulmonary Disease Tool (EXACT^®^) daily and COPD Assessment Test™ (CAT) and Patient Global Impression of Severity (PGIS) of COPD weekly on either PD or BYOD for 15 days, then switched device types for 15 days. EXACT was scored using the Evaluating Respiratory Symptoms in COPD (E-RS^®^: COPD) algorithm and equivalence assessed using intraclass correlation coefficients (ICCs) adjusting for cross-over sequence, period, and time. Two one-sided tests (TOSTs) used ICC adjusted means with 10%, 20%, and 40% of total score tested as equivalence margins. Compliance and comfort with technology were assessed. Equivalence across 3 device screen sizes was assessed following the second completion period.

**Results:**

Participants (N = 64) reported high comfort with technology, with 79.7% reporting being “quite a bit” or “very” comfortable. Weekly compliance was high (BYOD = 89.7–100%; PD = 76.9–100%). CAT and E-RS: COPD scores correlated well with PGIS (*r* > 0.50) and demonstrated equivalence between PD and BYOD completion (ICC = 0.863–0.908). TOST equivalence was achieved within 10% of the total score (*p* > 0.05). PRO measure scores were equivalent across 3 different screen sizes (ICC = 0.972–0.989).

**Conclusions:**

Measure completion was high and scores equivalent between PD and BYOD, supporting use of BYOD in addition to PD for collecting PRO data in COPD studies and in demographically diverse patient populations.

**Supplementary Information:**

The online version contains supplementary material available at 10.1186/s41687-022-00521-3.

## Highlights

- Historically, provisioned handheld devices [PD] have been provided to participants to enter patient-reported outcome (PRO) data during a clinical trial. Allowing participants to report data using their own smartphone or other internet-connected device (known as ‘bring your own device’ [BYOD]) is of growing interest.
- Measure completion was high for both device types when assessing daily and weekly compliance. Scores were found to be equivalent for both the Evaluating Respiratory Symptoms in COPD and COPD Assessment Test™ between PD and BYOD using 2 different methods. This study supports use of BYOD in addition to PD for collecting PRO data in COPD studies and contributes evidence that BYOD may be successfully employed in demographically diverse patient populations.

**Supplementary Information:**

The online version contains supplementary material available at 10.1186/s41687-022-00521-3.

## Background

Patient-reported outcome (PRO) measures assess disease-related symptoms and functional impacts, which, in a clinical trial setting, are needed for understanding the clinical benefit of drugs and other medical products from the patient’s perspective. Moreover, when studying a disease or condition which has a high degree of symptom variability, daily PRO measure collection is often required to capture this variability and obtain a representative picture of the experience of the people living with the disease. In the past, daily PRO data collection involved the participant completing a paper version of the PRO measure each day. Paper-based diary formats suffer many issues around completion and missing data, such as unanswered items and retrospective completion. To address such concerns, electronic formats of PRO measures (ePRO) have been developed which provide better control over measure completion through the use of alerts, reminders, and windows which restrict PRO data entry to a specified time of day, and thus prevent retrospective completion.

Modes of ePRO data collection have also evolved over time. Historically, handheld electronic devices were provided to participants to report PRO data in clinical trials and other research studies. These are known as provisioned devices (PD). However, the desire to reduce drug development costs and participant burden, combined with improved technology and greater access, has led to increasing interest in having participants use their own devices (‘bring your own device’ [BYOD]) to collect PRO data. As advances in technology have led to increased data security for storage and transfer of information for both PD and BYOD, the BYOD approach is becoming an increasingly viable option for large-scale, interventional trials. In addition, it is increasingly possible to ensure that the presentation of items is consistent regardless of the device type used. A growing body of evidence supports measurement comparability between paper and a variety of electronic formats, underlining the hypothesis that small presentational or format changes between devices have little to no impact on the integrity of PRO data [1].

Previous studies have explored the feasibility of using a BYOD approach for PRO data collection and have demonstrated high compliance with daily completion, high participant acceptance, and score equivalence between PD and BYOD for common response scale types [2–5]. However, they did not assess score equivalence and compliance between PD and BYOD in a longitudinal study design. Thus, the primary objective of this study was to quantitatively compare PRO data collected longitudinally via PD versus BYOD in terms of compliance, score agreement and equivalence. In addition, the secondary objective was to cross-sectionally evaluate the comparability of PRO scores collected on screens of varying sizes.

## Methods

### Participants

Participants with a clinical diagnosis of chronic obstructive pulmonary disease (COPD) were recruited from 4 clinical sites in the United States (US). Participants were screened on the basis of inclusion and exclusion criteria less restrictive than, but similar to, those likely to be used in a COPD clinical trial. Less restrictive criteria were used because this was a non-interventional study for which stable participants were being sought and no treatment effect was being evaluated. The criteria used in this study were similar to the criteria used in the development and testing of the EXACT^®^:

#### Inclusion criteria

1. Age ≥ 40 years
2. Established clinical diagnosis of COPD in accordance with the joint American Thoracic Society/European Society’s definition
3. Forced expiratory volume in one second (FEV_1_)/forced vital capacity ratio of < 0.70 post-bronchodilator
4. FEV_1_ of predicted < 80%
5. Current or former smoker with a history of at least 10 pack years
6. Clinical status and treatment unlikely to change in the next 30 days in the opinion of the investigator or referring clinician
7. Owns a compatible smartphone for the BYOD component of the study
8. Able to read, comprehend, and complete questionnaires and interviews in US English
9. Able to provide written informed consent given prior to undertaking any study-related procedures

#### Exclusion criteria

1. Had a COPD exacerbation, including hospitalization or hospitalization for pneumonia, within the previous 90 days
2. Professional involvement in or immediate family member of staff working on this study
3. Participated in any BYOD study within the previous 90 days
4. Had learning, emotional, mental illness, or cognitive difficulties that might limit ability to meaningfully complete the questionnaires
5. Showed evidence of alcohol or drug abuse

### Study design

**Fig. 1:**
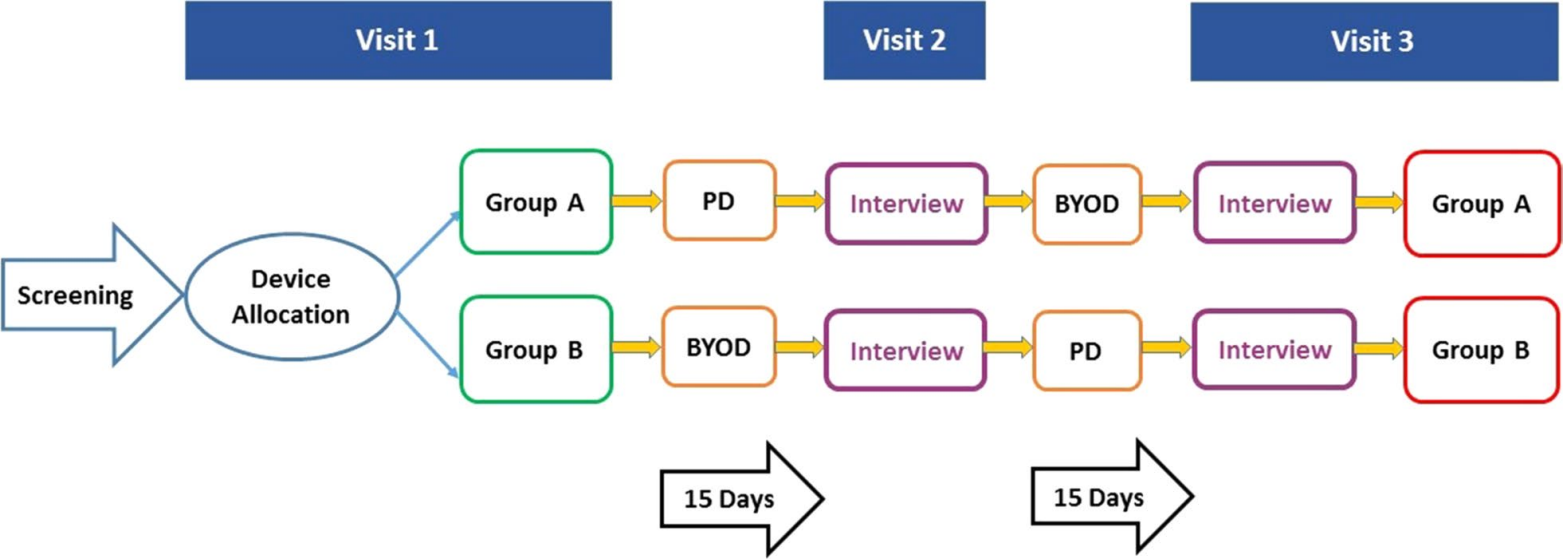
Study design. *Note* The full study design included interviews with all study participants, and the interview results are reported elsewhere [6]

In this observational, cross-over study (Fig. 1) participants were assigned to use one device type for 15 days (PD or BYOD) prior to switching to the other device type for an additional 15 days. Participants were split into 2 groups, Group A and Group B, and attended 3 study visits. At Visit 1, participants in Group A received the PD (Samsung Galaxy 4 running Android v4.4.4 with a 5″ touchscreen) with the software already installed, whereas participants in Group B installed the application on their own smartphone by downloading and activating the application (either Android or iOS) (Additional file 1: Fig. S1). Both groups completed a short training module on the respective device (Fig. 1). A 6-h completion window was set for each participant during this visit; this window was set to range from 3 h before to 3 h after the participant’s “usual” bedtime. It was not possible to complete the PRO measures outside of this 6-h window.

Following Visit 1, and for the next 15 days, participants completed 3 PRO measures on the device type they were allocated at Visit 1. The EXAcerbations of Chronic Pulmonary Disease Tool (EXACT^®^) was completed every day during the participant’s completion window. The COPD Assessment Test™ (CAT) was completed immediately after the EXACT^®^ every 7 days at the start, middle, and end of each 15-day data collection period (i.e., Days 1, 8, 15, of each cross-over period). The Patient Global Impression of Severity (PGIS) of COPD was completed after the EXACT^®^ and CAT and was also completed every 7 days at the start, middle, and end of each 15-day data collection period. A reminder notification from the ePRO system was sent to the participant’s device if they had not completed the EXACT^®^ at 1 h before their “usual” bedtime, at bedtime, and 1 h after bedtime. Participants were able to turn off reminders on their own device (although they were not shown how to do so), but not on the PD. Participants did not receive reminders in any other modality (e.g., telephone calls from site staff) to complete the EXACT^®^ or any other PRO measures included in the study.

At Visit 2, participants were switched to the other device type to complete the same PRO measures for an additional 15 days using the same schedule of assessment as per the data collection Period 1. At this visit, participants were interviewed about their experience with the first data collection period and were given training on how to use the second device.

At Visit 3, participants returned to the site to complete all study procedures including returning the PD for those in Group B and a final interview. A subset of 20 participants was invited to complete a screen size equivalence test during which the EXACT^®^ and CAT were completed on a provisioned smartphone, a provisioned tablet, and a provisioned laptop computer at the site with a distraction task (completion of a word search puzzle for approximately 30 min) between each device completion.

### Study measures

#### EXAcerbations of Chronic Pulmonary Disease Tool (EXACT^®^)

The EXACT^®^ is a 14-item patient-reported daily diary used to quantify and measure exacerbations of COPD [7]. It was scored using the Evaluating Respiratory Symptoms in COPD (E-RS^®^: COPD) algorithm, which derives a score from 11 items of the EXACT. It measures the effect of treatment on the severity of respiratory symptoms in stable COPD. In this study, the entire 14-item EXACT was administered per license requirements, however, only the 11-item E-RS: COPD was evaluated.

The E-RS: COPD produces a daily Total Score (RS-Total Score; scored from 0 to 40) and three subscales: RS-Breathlessness (5 items; scored from 0 to 17), RS-Cough and Sputum (3 items; scored from 0 to 11), and RS-Chest symptoms (3 items; scored from 0 to 12). A higher score on each domain indicates more severe respiratory symptoms. Daily total scores were computed using scoring rules specified in the user manual. Weekly total scores were calculated using the sum of daily scores divided by the number of diary days completed. A minimum of 4 out of 7 days of data was required to compute a weekly mean.

#### COPD assessment test (CAT)

The CAT comprises 8 questions that assess domains related to the impacts of COPD: cough, phlegm, chest tightness, breathlessness, activities at home, confidence to leave the home, sleep, and energy [8]. Each question is scored from 0 to 5, with higher scores indicating greater problems with the domain. A total score (0 to 40) is produced, with scores of 0 to 10, 11 to 20, 21 to 30, and 31 to 40 representing mild, moderate, severe, and very severe clinical impact, respectively.

#### Patient Global Impression of Severity (PGIS)

This single-item measure asks participants to report the perceived severity of their COPD symptoms over the previous 7 days on a 5-point scale from 0 (“none”) to 4 (“very severe”).

### Analyses

#### Compliance analysis

Daily and weekly compliance of the EXACT were evaluated and calculated by device type. Compliance for the CAT was evaluated at each key assessment time point (Day 1, 8, 15 of each cross-over period) and calculated by device type. For the EXACT, compliance was defined based on the number of missing and completed diary days in each weekly period according to device type. EXACT^®^ items could not be skipped, so only form-level compliance evaluation was performed on the measure as a whole. As CAT items could be skipped, item-level compliance for the CAT was calculated at each key assessment time point.

#### Equivalence analysis

- Descriptive statistics: Descriptive PRO measure scores between cross-over periods were calculated for top-level comparisons of equivalence.
- Score agreement of E-RS: COPD and CAT scores between device type: A linear mixed effects model, adjusting for cross-over sequence, period, and time (day/week) was used to derive the variance components used in the calculation of the intraclass correlation coefficients (ICC). An ICC_(2,1)_ model was used to assess the absolute agreement of scores arising from the 2 device types in this cross-over study [9]. The PGIS was included as an anchor measure to define the stable population (those whose score did not change between assessment time points) for these equivalence analyses.
- Equivalence between device type: Two one-sided test (TOST) analyses on both the unadjusted means and the adjusted means derived from the mixed effects model were conducted. The 90% confidence intervals (CIs) around the difference in score means between the devices was tested against an equivalence margin of 10%, 20%, and 40% of total score. This equated to equivalence margins of 4-points, 8-points and 16-points for both the E-RS: COPD and the CAT.
- Equivalence between screen size *(substudy analysis)*: For the CAT and E-RS: COPD scores, agreement for the 3 different screen sizes (PD [smartphone], tablet, and laptop) were assessed using the ICC_(2,1)_ model. Additionally, the mean scores, differences between the means for each of the screen sizes, as well as associated standard error of the mean and the 95% CIs were reported.

## Results

### Demographic and clinical characteristics

Sixty-four participants were enrolled (mean age [SD]: 59.0 [10.55]; 65.6% female; 51.6% Black/African American) (Table 1). Participants in Group A (n = 23) and Group B (n = 41) were of similar mean [SD] ages (Group A = 57.5 years [11.33]; Group B = 59.8 years [10.13]) and the gender distribution was also similar (Group A n = 14 female, 60.9%; Group B n = 28 female, 68.3%). Race distributions amongst the two groups did not reflect the overall study sample. Group A participants were more likely to be White (n = 13, 56.5%) whereas Group B participants were more likely to be Black or African American (n = 27, 65.9%).

**Table 1:** Demographic and clinical characteristics

Demographic or clinical item	Group A (PD 1st) n = 23	Group B (BYOD 1st) n = 41	Overall N = 64
Mean age (SD)	57.5 (11.33)	59.8 (10.13)	59.0 (10.55)
Range	40–75	40–77	40–77
Sex			
Female	14 (60.9%)	28 (68.3%)	42 (65.6%)
Male	9 (39.1%)	13 (31.7%)	22 (34.4%)
Race			
White	13 (56.5%)	13 (31.7%)	26 (40.6%)
Black/African American	6 (26.1%)	27 (65.9%)	33 (51.6%)
Other	4 (17.4%)	1 (2.4%)	5 (7.8%)
Ethnicity			
Hispanic/Latino	4 (17.4%)	1 (2.4%)	5 (7.8%)
Not Hispanic/Latino	18 (78.3%)	40 (97.6%)	58 (90.6%)
Do not wish to state	1 (4.8%)	0	1 (1.7%)
Education			
Did not complete high school	0	5 (12.2%)	5 (7.8%)
High school diploma	8 (34.8%)	12 (29.3%)	20 (31.3%)
Some college or certificate program	6 (26.1%)	13 (31.7%)	19 (29.7%)
College or university degree	9 (39.1%)	8 (19.5%)	17 (26.6%)
Graduate degree	0	3 (7.3%)	3 (4.7%)
Work status			
Employed full-time	15 (65.2%)	11 (26.8%)	26 (40.6%)
Employed part-time	2 (8.7%)	4 (9.8%)	6 (9.4%)
Homemaker	1 (4.3%)	1 (2.4%)	2 (3.1%)
Retired	2 (8.7%)	12 (29.3%)	14 (21.9%)
Unemployed	0	3 (7.3%)	3 (4.7%)
Disabled/on disability	3 (13.0%)	9 (22.0%)	12 (18.8%)
Other	0	1 (2.4%)	1 (1.6%)
Gold stage			
II	16 (69.6%)	30 (73.2%)	46 (71.9%)
III	6 (26.1%)	10 (24.4%)	16 (25.0%)
IV	1 (4.3%)	1 (2.4%)	2 (3.1%)
Participant-reported overall health			
Very good	3 (13.0%)	8 (19.5%)	11 (17.2%)
Good	16 (69.6%)	17 (41.5%)	33 (51.6%)
Fair	3 (13.0%)	15 (36.6%)	18 (28.1%)
Poor	1 (4.3%)	1 (2.4%)	2 (3.1%)
Clinician-reported COPD severity			
Very mild	0	1 (2.4%)	1 (1.6%)
Mild	0	13 (31.7%)	13 (20.3%)
Moderate	18 (78.3%)	18 (43.9%)	36 (56.3%)
Severe	5 (21.7%)	8 (19.5%)	13 (20.3%)
Very severe	0	1 (2.4%)	1 (1.6%)

*BYOD* Bring your own device, *COPD* Chronic obstructive pulmonary disease, *PD* Provisioned device

The overall sample contained participants who were diagnosed with COPD for an average of 7.2 years prior to enrollment, and half of the sample had no prior exacerbations (n = 32, 50.0%), with around a third of the sample reporting a single previous exacerbation (n = 20, 31.3%). Participants reported high comfort with technology in general, with 79.7% reporting being “quite a bit” or “very” comfortable (Additional file 1: Table S1). Descriptions for BYOD devices are included in Additional file 1: Table S2.

### Compliance

For the first 15 days of the study (data collection Period 1), almost all Group A participants (who were using PD) completed the EXACT on at least 5 days of Week 1 (95.2%; n = 20), compared with 89.7% (n = 35) of Group B participants (using BYOD) (Additional file 1: Figs. S2 and S3). Compliance in Week 2 followed a similar pattern but was slightly higher among participants in both groups. One Group B participant in Week 1 and 2 Group B participants in Week 2 did not complete the EXACT on any of the 7 days.

Following the device cross-over, in data collection Period 2, 100% of Group A participants (now using BYOD) completed the EXACT on at least 5 days in Weeks 1 and 2 of Period 2. Compliance among Group B participants (now using a PD) was lower than Group A participants, with 76.9% (n = 30) and 89.2% (n = 34) completing the EXACT on at least 5 days during Week 1 and 2, respectively. Three Group B participants in Week 1 and 4 Group B participants in Week 2 did not complete the EXACT on any of the respective 7 days. Additional file 1: Table S3 presents the distributions of percent days completed and percent of participants completing each week.

Given that participants were able to skip CAT items (with confirmation that skipping was intentional), item-level compliance was assessed at all key assessment time points in participants who had at least 1 CAT item, to identify any items that may have a non-random skip rate. High item-level compliance (94.1 to 100%) was found among participants who completed the CAT ( Additional file 1: Table S4). In Period 1, Items 3 (cough), 4 (breathlessness), 6 (confidence to leave home), and 7 (sleep) all showed some level of missing data. The missingness for these items was, however, minimal. Item compliance was higher in Period 2 of the study, where all participants achieved 100% item compliance.

### Equivalence: descriptive assessment

For each study week across all 4 weeks, participants’ overall mean [SD] E-RS: COPD scores were similar (14.73 [6.800]–15.28 [6.497]), suggesting score equivalence between devices. Table 2 shows the means for Group A and Group B independently. Means across the cross-over period remain similar within both groups.

**Table 2:** ERS-COPD and CAT equivalence descriptive statistics

Period	Time point	Statistic	Group A PD 1st (N = 21)	Group B BYOD 1st (N = 39)	Overall (N = 60)
*ERS-COPD equivalence descriptive statistics*					
Period 1	Week 1	n	21	35	56
		Mean (SD)	17.31 (5.961)	14.06 (6.580)	15.28 (6.497)
		Median	17.43	12.57	15.08
		Q1	13.57	10.00	10.14
		Q3	21.57	18.60	20.21
		Min, Max	6.7, 25.8	1.0, 28.7	1.0, 28.7
	Week 2	n	20	36	56
		Mean (SD)	16.96 (6.003)	13.58 (6.845)	14.79 (6.704)
		Median	17.36	13.46	15.54
		Q1	13.06	9.24	9.92
		Q3	21.37	16.36	19.04
		Min, Max	4.0, 27.6	0.4, 32.7	0.4, 32.7
Period 2 (device switch)	Week 1	n	21	32	53
		Mean (SD)	17.37 (5.755)	13.59 (6.456)	15.09 (6.410)
		Median	19.71	12.29	14.43
		Q1	13.57	9.85	10.17
		Q3	21.20	17.29	20.14
		Min, Max	5.9, 24.3	2.0, 31.0	2.0, 31.0
	Week 2	n	21	32	53
		Mean (SD)	17.57 (5.290)	12.87 (7.103)	14.73 (6.800)
		Median	19.00	11.89	15.67
		Q1	12.14	6.93	9.86
		Q3	21.57	18.71	19.71
		Min, Max	7.6, 24.9	0.0, 27.6	0.0, 27.6
*CAT equivalence descriptive statistics*					
Period 1	Day 1	n	20	27	47
		Mean (SD)	20.70 (8.880)	19.41 (7.360)	19.96 (7.975)
		Median	21.00	19.00	20.00
		Q1	13.00	12.00	13.00
		Q3	27.50	25.00	27.00
		Min, Max	3.0, 37.0	8.0, 33.0	3.0, 37.0
	Day 8	n	18	36	54
		Mean (SD)	23.11 (7.451)	20.81 (8.263)	21.57 (8.006)
		Median	26.00	21.50	23.00
		Q1	17.00	14.50	15.00
		Q3	29.00	25.50	28.00
		Min, Max	7.0, 32.0	5.0, 37.0	5.0, 37.0
	Day 15	n	20	30	50
		Mean (SD)	22.85 (6.753)	20.07 (7.904)	21.18 (7.521)
		Median	24.00	22.00	23.00
		Q1	20.50	12.00	15.00
		Q3	28.00	26.00	27.00
		Min, Max	8.0, 32.0	5.0, 34.0	5.0, 34.0
Period 2 (device switch)	Day 1	n	18	32	50
		Mean (SD)	23.39 (8.001)	18.19 (8.506)	20.06 (8.622)
		Median	24.50	18.00	20.00
		Q1	18.00	11.00	12.00
		Q3	29.00	24.00	26.00
		Min, Max	10.0, 38.0	4.0, 34.0	4.0, 38.0
	Day 8	n	18	30	48
		Mean (SD)	19.94 (8.120)	19.53 (7.908)	19.69 (7.904)
		Median	22.00	20.50	21.00
		Q1	14.00	11.00	13.00
		Q3	26.00	25.00	25.50
		Min, Max	6.0, 33.0	7.0, 33.0	6.0, 33.0
	Day 15	n	20	29	49
		Mean (SD)	19.95 (7.273)	20.52 (7.317)	20.29 (7.228)
		Median	21.00	20.00	21.00
		Q1	15.50	15.00	15.00
		Q3	24.00	27.00	26.00
		Min, Max	8.0, 33.0	7.0, 34.0	7.0, 34.0

Program: (t_act.sas) (07FEB18:16:40:23)

Analysis datasets: _adpro

*BYOD* Bring your own device, *CAT* Chronic obstructive pulmonary disease (COPD) assessment test, *CSP* Cross sectional population, *PD* Provisioned device

The CAT is an 8-item self-assessment measure with an additive total score. A higher score on the CAT relates to increased severity. Participants could skip CAT items

The PD 1st Device Group consists of all participants who started the study using the provisioned device and the BYOD 1st Group consists of all participants who started the study using the BYOD. Groups display participants based on the device that they used for the first 15-day period of the trial, not necessarily the device they were initially randomized to. Both groups switched device types at Period 2 Day 1

The CSP consists of all participants who were enrolled in the study and are still enrolled at the specified time point

Overall, CAT scores between Period 1 and Period 2 (Days 1, 8, and 15, respectively) were similar (Table 2). For Group A and Group B independently, means between the 2 cross-over periods showed minor differences with Group B showing a lower mean after switching to the second device type at Period 2 Day 1 while Group A showed a higher mean at Period 2 Day 1 (Group A: Period 1 Day 1 = 20.7 [8.88], Period 2 Day 1 = 23.4 [8.00]; Group B: Period 1 Day 1 = 19.4 [7.36], Period 2 Day 1 = 18.2 [8.51]).

### Device order

Information on order preference collected in this participant population and presented in Newton et al. showed that participants preferred their second device [6]. Specifically, participants who started the study on the BYOD device preferred the PD (36.36%) and participants who started the study on the PD device preferred the BYOD device (30.91%; *p* = 0.0076). Based on this analysis, the TOST and ICC estimations were derived with the inclusion of device in each model.

### Equivalence: ICC based

When E-RS: COPD weekly and CAT scores were adjusted for order of device completion, study period, and assessment time point in a full cross-over design using a mixed effects model, agreement across device types was high for the E-RS: COPD weekly average score (ICC(2,1) = 0.878) and the CAT score (ICC(2,1) = 0.864), see Table 3. For Stable Population 1 (SP1) defined as participants who indicated no change from baseline to Day 30 on the PGIS, consistency across device types was even higher for the E-RS: COPD with ICC(2,1) = 0.895. Stable Population 2 (SP2), defined as participants who indicated minimal (|1|) to no change from baseline to Day 30 on the PGIS, had high reliability but with the slightly lower ICC reflecting the increased variance expected with this sample; ICC(2,1) = 0.863.

**Table 3:** ICC cross-over

Endpoint	Population	N	ICC
E-RS: COPD total^a^	LPP^c^	58	0.878
	SP1^c^	22	0.895
	SP2^c^	41	0.863
CAT total^b^	LPP^c^	45	0.864
	SP1^c^	21	0.908
	SP2^c^	40	0.874

Program: (t_icc_crossover.sas) (31MAY18:15:49:40)

Analysis datasets: _adpro

*BYOD* Bring your own device, *CAT* Chronic obstructive pulmonary disease (COPD) assessment test, *E-RS: COPD* Evaluating respiratory symptoms in COPD, *LPP* Longitudinal period population, *PD* Provisioned device, *SP1* Stable population 1, *SP2* Stable population 2

^a^The E-RS: COPD is a total score derived from 11 items of the 14-item EXACT^®^. It is scored as per the missing data and scoring rule specified in the user manual. A higher score on the E-RS: COPD relates to increased severity of respiratory condition

^b^The CAT is an 8-item self-assessment measure with an additive total score. A higher score on the CAT relates to increased severity. Participants could skip CAT items

^c^The LPP was defined for each PRO measure from Day 1 to each of the following assessment periods. Any participant who was maintained in the study into each assessment day from Day 1 to the analysis-specified subsequent assessment day and had PRO data available for that time point was included. The LPP was further delineated with 2 additional population flags for identifying stable participants for the evaluation of measurement equivalence: Stable Population 1 (SP1) included participants who indicated no change from baseline to Day 30 on the PGIS and Stable Population 2 (SP2) included participants who indicated minimal (|1|) to no change from baseline to Day 30 on the PGIS

Similarly, high agreement statistics were determined for the CAT when assessed in a full cross-over design using the SP1 (ICC(2,1) = 0.908) and SP2 (ICC(2,1) = 0.874) populations. The CAT scores were also consistent between devices on Day 15 of Period 1 and Day 1 of Period 2 (Day 16), with ICC(2,1) = 0.836. These two days were selected because they were approximately 24 h apart, which reduced the chance of a change in the participant’s COPD status when moving from one device type to the next.

### Equivalence: TOSTs

When analyses were conducted using a full cross-over design using all available data and accounting for order of device completion, study period, and assessment time point, TOST analysis showed that the 2 device types were equivalent for both the E-RS: COPD and the CAT scores. Ninety percent (90%) CIs around the differences between scores on the 2 device types fell within the ± 10% equivalence levels. This result shows that both measures were equivalent within a ± 10% (4-point) equivalence margin (Table 4).

**Table 4:** TOSTs cross-over

Endpoint	Population	N		LS mean difference (SE)	90% CI
ERS™: COPD total^a^	LPP^c^	58	PD–BYOD	− 0.64 (0.362)	− 1.237, − 0.039
	SP1^c^	22	PD–BYOD	− 1.42 (0.431)	− 2.137, − 0.699
	SP2^c^	41	PD–BYOD	− 0.75 (0.373)	− 1.373, − 0.136
CAT total^b^	LPP^c^	45	PD–BYOD	− 0.14 (0.471)	− 0.913, 0.642
	SP1^c^	21	PD–BYOD	− 1.99 (0.681)	− 3.119, − 0.857
	SP2^c^	40	PD–BYOD	− 0.26 (0.514)	− 1.112, 0.588

Program: (t_icc_crossover.sas) (31MAY18:15:49:40)

Analysis datasets: _adpro

*BYOD* Bring your own device, *CAT* Chronic obstructive pulmonary disease (COPD) assessment test, *E-RS: COPD* Evaluating respiratory symptoms in COPD, *LPP* Longitudinal period population, *PD* Provisioned device, *SP1* Stable population 1, *SP2* Stable population 2

^a^The E-RS: COPD is a total score derived from 11 items of the 14-item EXACT^®^. It is scored as per the missing data and scoring rule specified in the user manual. A higher score on the E-RS: COPD relates to increased severity of respiratory condition

^b^The CAT is an 8-item self-assessment measure with an additive total score. A higher score on the CAT relates to increased severity. Participants could skip CAT items

^c^The LPP was defined for each PRO measure from Day 1 to each of the following assessment periods. Any participant who was maintained in the study into each assessment day from Day 1 to the analysis-specified subsequent assessment day and had PRO data available for that time point was included. The LPP was further delineated with two additional population flags for identifying stable participants for the evaluation of measurement equivalence: Stable Population 1 (SP1) included participants who indicated no change from baseline to Day 30 on the PGIS and Stable Population 2 (SP2) included participants who indicated minimal (|1|) to no change from baseline to Day 30 on the PGIS

### Screen size equivalence (substudy)

Both the E-RS: COPD and CAT measures performed consistently across the 3 device screen sizes, with high ICCs reported for both the E-RS: COPD (ICC(2,1) = 0.989) and the CAT (ICC(2,1) = 0.972), see Table 5.

**Table 5:** Screen size substudy descriptive statistics

Device	n	Mean (SD)	SE	95% CI
CAT^a^				
Laptop	19	17.8 (8.61)	1.98	(13.69, 21.99)
Smartphone	20	17.8 (9.11)	2.04	(13.49, 22.01)
Tablet	20	17.9 (8.98)	2.01	(13.70, 22.10)
Laptop versus Smartphone	19	0.1 (2.69)	0.62	(− 1.19, 1.40)
Laptop versus Tablet	19	− 0.1 (1.81)	0.42	(− 0.92, 0.82)
Smartphone versus Tablet	20	− 0.2 (1.81)	0.41	(− 1.00, 0.70)
E-RS: COPD^a^				
Laptop	20	22.0 (7.75)	1.73	(18.37, 25.63)
Smartphone	20	22.0 (7.62)	1.70	(18.43, 25.57)
Tablet	20	22.1 (8.06)	1.80	(18.28, 25.82)
Laptop versus Smartphone	20	0.0 (1.03)	0.23	(− 0.48, 0.48)
Laptop versus Tablet	20	− 0.1 (1.28)	0.29	(− 0.65, 0.55)
Smartphone versus Tablet	20	− 0.1 (1.32)	0.29	(− 0.67, 0.57)

Program: (t_icc_screen_anova.sas) (12FEB18:09:56:52)

Analysis datasets: _adpro

*CI* Confidence interval, *E-RS: COPD* Evaluating respiratory symptoms in COPD, *SD* Standard deviation, *SE* Standard error

^a^The E-RS: COPD is a total score derived from 11 items of the 14-item EXACT^®^. It is scored as per the missing data and scoring rule specified in the user manual. A higher score on the E-RS: COPD relates to increased severity of respiratory condition. The CAT is an 8-item self-assessment measure with an additive total score. A higher score on the CAT relates to increased severity. Participants could skip CAT items

Mean differences between the different screen sizes were also assessed for the E-RS: COPD and CAT. The differences and associated 95% CIs support the ICC results in showing that participants responded consistently across all device screen sizes.

## Discussion

There were some limitations to the study. Device type assignment was not randomized due to technical issues with PDs during enrollment (see Newton et al. [6] for more details*)*, which led to differences between Group A and Group B in terms of unequal sample size and demographics that appeared to impact the analysis of compliance. Therefore, the study was not able to answer the question of whether compliance is better with PD or BYOD because of many confounding factors. In the second part of this cross-over study, there was an increase in the number of participants (n = 7/41; 17%) missing the entire week in Group B after switching to PD; Group A compliance improved in Period 2. We were not able to determine the cause for the poorer compliance in Period 2 for Group B. In addition, subgroup analyses revealed a possible order effect in that participants preferred the device they used in the second study period. Therefore, some planned analyses regarding device compliance by subgroup were not conducted due to this order effect. As a result, we concluded that compliance was adequate using both the PD and BYOD approaches but did not find one better than the other. In the screen size equivalence test, the time interval between completing the 3 different screen sizes was short, which may have contributed to the resulting high ICC. The study included only participants diagnosed with COPD because the content of the PRO measures included in the study was relevant to them; therefore, the study results may not be generalizable to respondents with other diagnoses.

This study found that diary completion was high for both device types when assessing daily compliance as well as a threshold for the weekly assessment of 5 or more days of complete diary data, and neither device type was better nor worse than the other. A key aim of this study was to assess score equivalence of data obtained from the 2 device types. Descriptively, the overall mean scores for the E-RS: COPD and the CAT before and after the cross-over period were very similar between device types, though means for Group B were slightly lower, reflecting a less severe sample. Group B was also less compliant in both study periods. However, despite the minor differences in the group means, the TOST analysis showed that the difference of scores fell within a 20% margin at the majority point and ICC showed that the score concordance between the two device types was very high for the E-RS™: COPD weekly average score and the CAT. Post hoc tests, which adjusted for the cross-over design of the study, showed high ICCs for COPD measure scores between the 2 device types and that scores on both the E-RS™: COPD and the CAT were equivalent at the 10% level between PD and BYOD. Overall, the evidence supports score equivalence between the 2 device types, even when considering the additional variance introduced by the unequal sample sizes. Score equivalence was demonstrated across both the PD and BYOD devices with CIs around the mean score differences for all measures within predefined ranges for equivalence. The screen size equivalence substudy using the E-RS™: COPD and CAT and completed on a PD, tablet, and laptop on the same day demonstrated that when mean scores were assessed between device pairs, mean scores and standard errors were similar between the devices and confidence intervals for mean differences were contained within the ± 10% equivalence acceptance intervals. The quantitative evidence, which met each of the predefined criteria, as well as the qualitative interview data (presented in Newton et al. [6]), which reported that participants felt comfortable with and did not indicate a clear preference for either device, suggests support for cross-platform equivalence of these PRO measures.

## Conclusions

The COPD population was chosen intentionally, given the severity of this chronic illness and typical age of persons who have it (average age 65 years). If PRO data and compliance data are similar within this population, these results could help support use of BYOD in less severe participants across a wider age range in future studies. Future research may also be needed to evaluate BYOD for event-driven diaries (e.g., migraine headaches, bowel movements) which our study did not address. This study supports the use of BYOD as a potential addition to PD for collecting PRO data in COPD studies and contributes evidence that BYOD may be employed to collect PRO data in demographically diverse patient populations. The findings from this study should encourage continued use and testing of BYOD in clinical studies, including evaluation in clinical trials, with the ultimate aim of providing study participants and sponsors with expanded remote data collection options.

## Supplementary Information

**Additional file 1. Table S1.** Participant experience with technology. **Table S2.** Participant BYOD smartphone description summary. **Table S3.** Number of missing days of diary data. **Table S4.** Item level compliance for the CAT. **Fig. S1.** Provisioned device application. **Fig. S2.** Number of missing days of EXACT completions in period 1 (weeks 1 and 2). **Fig. S3.** Number of missing days of EXACT completions in period 2 (weeks 1 and 2).

## Data Availability

The datasets generated and/or analyzed during the current study are not publicly available, but are available from the corresponding author on reasonable request.

## Abbreviations

BYOD: Bring your own device
CAT: COPD assessment test
CI: Confidence intervals
COPD: Chronic obstructive pulmonary disease
ePRO: Electronic formats of PRO
E-RSCOPD: Evaluating respiratory symptoms in COPD
EXACT: EXAcerbations of Chronic Pulmonary Disease Tool
FEV_1_: Forced expiratory volume in one second
ICC: Intraclass correlation coefficient
PD: Provisioned device
PGIS: Patient Global Impression of Severity
PRO: Patient-reported outcome
SP1: Stable population 1
SP2: Stable population 2
TOST: Two one-sided tests
US: United States
