# Supplementary material for: Comparability of a provisioned device versus bring your own device for completion of patient-reported outcome measures by participants with chronic obstructive pulmonary disease: quantitative study findings
Source: J Patient Rep Outcomes. 2022 Nov 26;6:119. doi: 10.1186/s41687-022-00521-3 (PMC9701291; doi:10.1186/s41687-022-00521-3)
Supplement: Supplementary file 1 — Additional file 1. Table S1. Participant experience with technology. Table S2. Participant BYOD smartphone description summary. Table S3. Number of missing days of diary data. Table S4. Item level compliance for the CAT. Fig. S1. Provisioned device application. Fig. S2. Number of missing days of EXACT completions in period 1 (weeks 1 and 2). Fig. S3. Number of missing days of EXACT completions in period 2 (weeks 1 and 2). [file 41687_2022_521_MOESM1_ESM.docx]

**Title:** Comparability of Provisioned Device Versus Bring Your Own Device for Completion of Patient-Reported Outcome Measures by Participants with Chronic Obstructive Pulmonary Disease: Quantitative Study Findings

**SUPPLEMENTARY MATERIALS (Tables and Figures)**

Table 1. Participant Experience with Technology

| **Technology Item** | **Group A (PD 1^st^) N=23** | **Group B (BYOD 1^st^)  N=41** | **Overall N=64** |
| --- | --- | --- | --- |
| **Comfort Using Own Smart Phone** | | | |
| Not At All Comfortable | 0 | 1 (2.4%) | 1 (1.6%) |
| A Little Bit Comfortable | 1 (4.3%) | 3 (7.3%) | 4 (6.3%) |
| Somewhat Comfortable | 0 | 8 (19.5%) | 8 (12.5%) |
| Quite A Bit Comfortable | 5 (21.7%) | 6 (14.6%) | 11 (17.2%) |
| Very Much Comfortable | 17 (73.9%) | 23 (56.1%) | 40 (62.5%) |
| **Comfort Using Technology** | | | |
| Not At All Comfortable | 1 (4.3%) | 5 (12.2%) | 6 (9.4%) |
| A Little Bit Comfortable | 2 (8.7%) | 3 (7.3%) | 5 (7.8%) |
| Somewhat Comfortable | 2 (8.7%) | 11 (26.8%) | 13 (20.3%) |
| Quite A Bit Comfortable | 3 (13.0%) | 7 (17.1%) | 10 (15.6%) |
| Very Much Comfortable | 15 (65.2%) | 15 (36.6%) | 30 (46.9%) |
| **Smartphone Type** | | | |
| Apple iOS | 10 (43.5%) | 13 (31.7%) | 23 (35.9%) |
| Android OS | 13 (56.5%) | 28 (68.3%) | 41 (64.1%) |
| BYOD - bring your own device; PD - provisioned device | | | |

Table 2. Participant BYOD smartphone description summary

| **Smartphone** | **Screen resolution** | **Screen size** | **Overall N=64** |
| --- | --- | --- | --- |
| **iOS (n=23)** |  |  |  |
| iPhone 5 / 5c | 640x1136 | 4-inch | 2 (3.1%) |
| iPhone 6 / 6s | 750x1334 | 4.7-inch | 11 (17.2%) |
| iPhone 7 | 750x1334 | 4.7-inch | 5 (7.8%) |
| iPhone 7 plus | 1080 x 1920 | 5.5-inch | 1 (1.6%) |
| Model not determined | - | - | 4 (6.2%) |
| **Android (n=41)** |  |  |  |
| LG G4 | 1440x2560 | 5.5-inch | 1 (1.6%) |
| LG K7 | 480x854 | 5-inch | 2 (3.1%) |
| Motorola Moto G4 | 1080x1920 | 5.5-inch | 2 (3.1%) |
| Samsung Note 5 | 1440x2560 | 5.7-inch | 1 (1.6%) |
| Samsung Prevail 2 | 480x800 | 4-inch | 1 (1.6%) |
| Make/model not determined | - | - | 34 (53.1%) |
|  | | | |

**Table 3. Number of Missing Days of Diary Data**

|  | | | | | | | |
| --- | --- | --- | --- | --- | --- | --- | --- |
|  | | | | | | | |
|  |  |  |  |  |  |  |  |
| _______________________________________________________________________________________________________________________________________________ | | | | | | |  |
|  | | | **PERIOD 1** | | **PERIOD 2** | |  |
| **Device** | | **Number of Missing Days (% Days Completed)** | **Week 1 Day 1-7** | **Week 2 Day 8-14** | **Week 1 Day 1-7** | **Week 2 Day 8-14** |  |
| **BYOD First** | | 0 (100%) | 23 (59.0%) | 24 (61.5%) | 21 (53.8%) | 24 (61.5%) |  |
|  | | 1 (86%) | 5 (12.8%) | 10 (25.6%) | 3 (7.7%) | 6 (15.4%) |  |
|  | | 2 (71%) | 7 (17.9%) | 3 (7.7%) | 6 (15.4%) | 4 (10.3%) |  |
|  | | 3 (57%) | 0 | 0 | 5 (12.8%) | 1 (2.6%) |  |
|  | | 4 (43%) | 1 (2.6%) | 0 | 0 | 0 |  |
|  | | 5 (28%) | 1 (2.6%) | 0 | 0 | 0 |  |
|  | | 6 (14%) | 1 (2.6%) | 0 | 1 (2.6%) | 0 |  |
|  | | 7 (0%) | 1 (2.6%) | 2 (5.1%) | 3 (7.7%) | 4 (10.3%) |  |
|  | | | | | | |  |
| **Provisioned First** | | 0 (100%) | 15 (71.4%) | 17 (81.0%) | 15 (71.4%) | 16 (76.2%) |  |
|  | | 1 (86%) | 4 (19.0%) | 3 (14.3%) | 6 (28.6%) | 3 (14.3%) |  |
|  | | 2 (71%) | 1 (4.8%) | 1 (4.8%) | 0 | 2 (9.5%) |  |
|  | | 3 (57%) | 1 (4.8%) | 0 | 0 | 0 |  |
|  | | 4 (43%) | 0 | 0 | 0 | 0 |  |
|  | | 5 (28%) | 0 | 0 | 0 | 0 |  |
|  | | 6 (14%) | 0 | 0 | 0 | 0 |  |
|  | | 7 (0%) | 0 | 0 | 0 | 0 |  |
|  | | | | | | |  |
| Program: (f_compliance.sas) (22FEB18:12:00:01)  Analysis datasets: _adpro | | | | | | |  |

**Table 4. Item Level Compliance for the CAT**

|  | **Day** | **Item 1** | **Item 2** | **Item 3** | **Item 4** | **Item 5** | **Item 6** | **Item 7** | **Item 8** |
| --- | --- | --- | --- | --- | --- | --- | --- | --- | --- |
| **Group A** | **Period 1** |  |  |  |  |  |  |  |  |
|  | 1 | 20 (100.0%) | 20 (100.0%) | 20 (100.0%) | 19 (95.0%) | 20 (100.0%) | 20 (100.0%) | 19 (95.0%) | 20 (100.0%) |
|  | 8 | 18 (100.0%) | 18 (100.0%) | 18 (100.0%) | 18 (100.0%) | 18 (100.0%) | 17 (94.4%) | 18 (100.0%) | 18 (100.0%) |
|  | 15 | 21 (100.0%) | 21 (100.0%) | 21 (100.0%) | 21 (100.0%) | 21 (100.0%) | 21 (100.0%) | 21 (100.0%) | 21 (100.0%) |
|  | **Period 2 (Device Switch)** |  |  |  |  |  |  |  |  |
|  | 1 | 17 (100.0%) | 17 (100.0%) | 17 (100.0%) | 17 (100.0%) | 17 (100.0%) | 17 (100.0%) | 17 (100.0%) | 17 (100.0%) |
|  | 8 | 14 (100.0%) | 14 (100.0%) | 14 (100.0%) | 14 (100.0%) | 14 (100.0%) | 14 (100.0%) | 14 (100.0%) | 14 (100.0%) |
|  | 15 | 16 (100.0%) | 16 (100.0%) | 16 (100.0%) | 16 (100.0%) | 16 (100.0%) | 16 (100.0%) | 16 (100.0%) | 16 (100.0%) |
| **Group B** | **Period 1y** |  |  |  |  |  |  |  |  |
|  | 1 | 30 (100.0%) | 30 (100.0%) | 30 (100.0%) | 30 (100.0%) | 30 (100.0%) | 29 (96.7%) | 30 (100.0%) | 30 (100.0%) |
|  | 8 | 36 (100.0%) | 36 (100.0%) | 36 (100.0%) | 36 (100.0%) | 36 (100.0%) | 36 (100.0%) | 36 (100.0%) | 36 (100.0%) |
|  | 15 | 34 (100.0%) | 34 (100.0%) | 33 (97.1%) | 32 (94.1%) | 34 (100.0%) | 33 (97.1%) | 34 (100.0%) | 34 (100.0%) |
|  | **Period 2 (Device Switch)** |  |  |  |  |  |  |  |  |
|  | 1 | 26 (100.0%) | 26 (100.0%) | 26 (100.0%) | 26 (100.0%) | 26 (100.0%) | 26 (100.0%) | 26 (100.0%) | 26 (100.0%) |
|  | 8 | 23 (100.0%) | 23 (100.0%) | 23 (100.0%) | 23 (100.0%) | 23 (100.0%) | 23 (100.0%) | 23 (100.0%) | 23 (100.0%) |
|  | 15 | 21 (100.0%) | 21 (100.0%) | 21 (100.0%) | 21 (100.0%) | 21 (100.0%) | 21 (100.0%) | 21 (100.0%) | 21 (100.0%) |
|  | | | | | | | | | |
| Abbreviations: BYOD - bring your own device; PD - provisioned device; CAT - Chronic Obstructive Pulmonary Disease (COPD) Assessment Test; CSP - cross sectional population. [1] The CAT is an 8 item self-assessment measure with an additive total score. A higher score on the CAT relates to increased severity. Participants could skip CAT items. [2] Group A consists of all participants who started the study using the provisioned device and the Group B consists of all participants who started the study using the BYOD. Groups display participants based on the device that they used for the first 15-day period of the trial, not necessarily the device they were initially randomized to. Both groups switched device types at Period 2 Day 1. [3] The CSP consists of all participants who were enrolled in the study and are still enrolled at the specified time point. Program: (t_completion_cat.sas) (karen.horton: 04APR18:11:31:12) Analysis datasets: _adpro | | | | | | | | | |

**Figure 1. Provisioned Device Application**

**INTRODUCTION**

The Exco Application is used to remind you when you have new in-app messages and when questionnaires can be completed. Below is information regarding how to use the phone and app.

**OPERATING THE PHONE**

| Power button  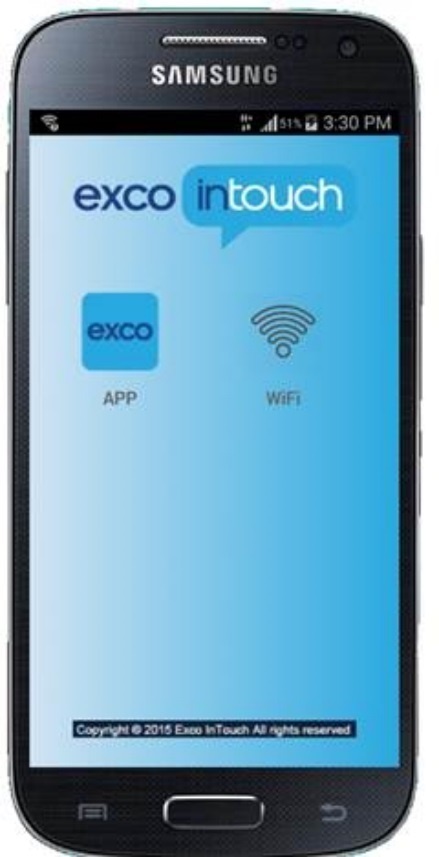  Home key  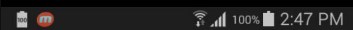 | Turn the phone on by pressing and holding the power button for 3 seconds. Release the power button and the phone will turn on within 30 seconds. The screen will light up when it is turned on. Turn the phone off by pressing and holding the same power button that you used to turn the phone on.  Control the phone by touching the icons (pictures) on the screen with your finger. You can make a selection by tapping once on the icon or button on the screen. To return to the home screen, press the “Home Key”. If “Home Key” is accidentally pressed while completing a diary, the App icon can be selected to return to the diary.  The battery status is on the top, right-hand side of the screen. When the icon is solid: the phone is charged. When the icon is red: the phone needs to be charged. Use the provided charger to charge the phone. Charge the phone each night so the battery is always charged. |
| --- | --- |

**CREATE A PIN**

| 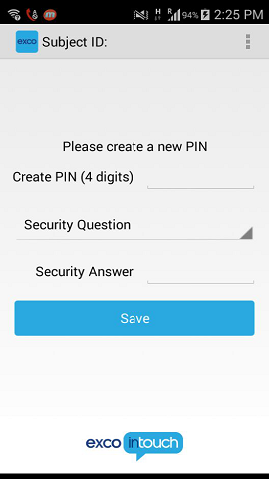  **Create A Pin** | To create a PIN, tap the blank space beside ‘Create PIN.’ This will allow you to enter a 4 digit pin.  Once entered, select a question from the ‘Security Questions’ list and then enter its answer in the ‘Security Answer’ blank. Tap Save to save all your responses in the application.  This will allow you to reset your pin if you ever need to, by entering the answer in the application. |
| --- | --- |

**LOG IN**

| 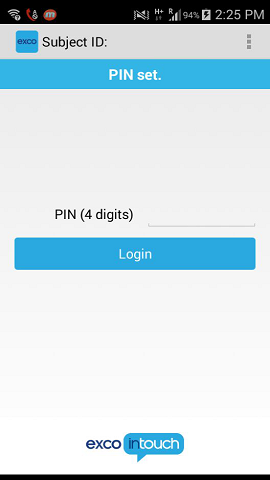  **Login Screen** | On the login screen, tap the blank area to the right of the label PIN(4 digits). Enter your PIN and tap LOGIN.  You have 3 attempts to successfully login to the application with your PIN. If you enter an incorrect PIN more than 3 times, you will be prompted to enter your security question and create a new PIN. |
| --- | --- |

**INSIDE THE APP**

| Messages Icon  Help Icon  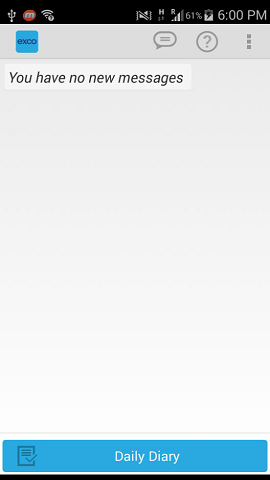  **App Home Screen** | The diary completion window will be set to open 3 hours prior to your average bedtime and will close 3 hours after that time. An alarm will be set within the app to remind you to complete the questionnaires one hour before bedtime, at bedtime, and one hour after bedtime if you have not yet completed them for the day.  Every time you log into the application, it will take you to the screen that has the ‘Daily Diary’ at the bottom, messages and help icon at the top.  When it is time to enter a daily diary, the icon for the diary will turn ‘blue’ at the bottom of the screen.  Tap the Daily Diary button to complete the questions each night. |
| --- | --- |

| 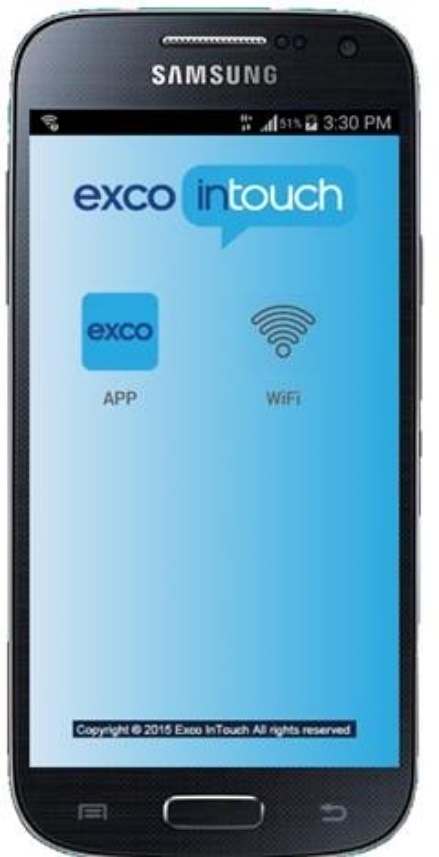 |
| --- |

**Figure 2. Number of Missing Days of EXACT Completions in Period 1 (Weeks 1 and 2)**

**
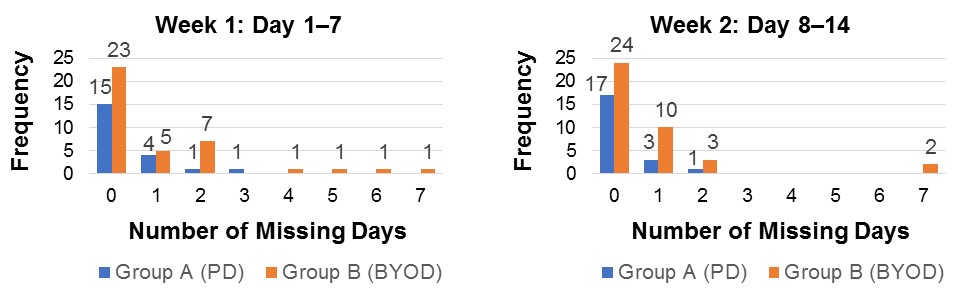
**

**Figure 3. Number of Missing Days of EXACT Completions in Period 2 (Weeks 1 and 2)**

**Week 1: Day 1-7**

**Week 2: Day 8-14**

**
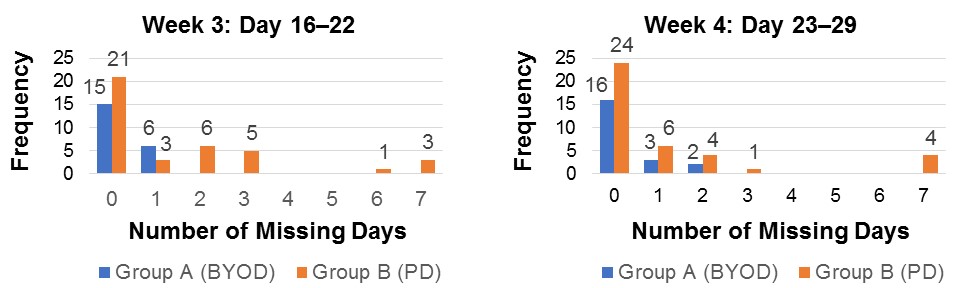
**
